# SARS‐CoV‐2 booster effect and waning immunity in hemodialysis patients: A cohort study

**DOI:** 10.1002/hsr2.1040

**Published:** 2023-01-10

**Authors:** Eibhlin Goggins, Binu Sharma, Jennie Z. Ma, Jitendra Gautam, Brendan Bowman

**Affiliations:** ^1^ Division of Nephrology University of Virginia School of Medicine Charlottesville Virginia USA; ^2^ Department of Public Health Sciences University of Virginia School of Medicine Charlottesville Virginia USA

**Keywords:** booster, COVID‐19 vaccination, end‐stage kidney disease, hemodialysis, humoral immunity, SARS‐CoV‐2

## 1 INTRODUCTION

Patients with end‐stage kidney disease on dialysis suffer high morbidity and mortality from severe acute respiratory syndrome coronavirus 2 (SARS‐CoV‐2). Despite successful vaccination campaigns by dialysis providers, the standard two‐dose vaccination series with Pfizer BioNTech (BNT162b2) messenger RNA (mRNA) SARS‐CoV‐2 is insufficient to protect patients from infection due to Omicron variants.^1^ Current guidelines recommend boosters of SARS‐CoV‐2 mRNA‐based vaccines.^2^ However, data regarding humoral response post‐booster is limited in dialysis patients. Additionally, few studies directly compare the long‐term response after two doses of a coronavirus disease 2019 (COVID‐19) vaccine to the response after three doses in the same cohort of patients. Studies suggest that the third dose of BNT162b2 increases antibody levels in dialysis patients.^3^ However, antibody response and booster effectiveness are diminished in dialysis patients compared with the general healthy population.^4^ We previously reported long‐term humoral responses to two doses of BNT162b2 in a cohort of hemodialysis patients.^5^ Six months after full vaccination, 40% of patients' anti‐spike protein IgG levels were either undetectable or borderline. Here, we report responses to the first booster of the BNT162b2 vaccine in these patients.

## 2 METHODS

We performed a prospective cohort study measuring serial semi‐quantitative IgG antibodies to the SARS‐CoV‐2 spike protein S1 receptor binding domain. We evaluated the response at a mean of 2, 6, and 11 weeks post‐booster. The Anti‐SARS‐CoV‐2 QuantiVac ELISA (IgG) from Euroimmun (EUROIMMUN US, Inc.) was used in all assessments. Final results were reported in WHO‐recommended binding antibody units (BAU/ml) per the manufacturer's instructions.^6^ Final results were considered negative for <25.6 BAU/ml, borderline for 25.6 to <35.2 BAU/ml, and positive for ≥35.2 BAU/ml. Clinical data were obtained as previously described.^5^

Of 35 hemodialysis patients in the original cohort, 27 (77.1%) received a third dose of BNT162b2, and 20/27 (74%) had complete data (4‐time point measurements): pre‐booster (mean of 6 weeks pre‐booster) and 2, 6, and 11 weeks post‐booster. Two weeks was used to attain peak/initial antibody levels.^7^ Subsequent samples were then obtained on a monthly basis when patients were scheduled for regular dialysis labs. Differences in antibody levels over time were compared nonparametrically using the Friedman test and then tested pairwisely with Bonferroni correction at alpha of <0.05. A linear mixed model was used to estimate decline in slope after vaccination.

## 3 RESULTS AND DISCUSSION

**Figure 1:**
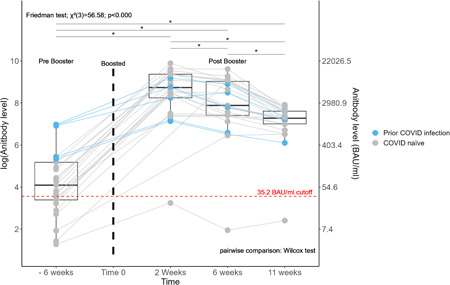
Trend in antibody level over time after a SARS‐COV‐2 BNT162b2 booster. Antibody levels were measured before and at an average of 2, 6, and 11 weeks after a SARS‐COV‐2 BNT162b2 booster vaccine in 27 patients. Each dot represents a subject and each line represents a single patient trend. The center line of each box plot represents the median. The lower and upper lines represent the 25th and 75th quartile, respectively. Friedman test was used for overall testing and Wilcoxon's signed rank test for pairwise comparison, as illustrated. *Represents a statistically significant difference in antibody level between two time points. Points under the red dashed line were below the borderline/negative cutoff for antibody level protection. SARS‐CoV‐2, severe acute respiratory syndrome coronavirus 2.

Antibody levels were significantly different over time (*χ* ^2^(3) = 56.6, *p* < 0.0001) from pre‐booster to 11 weeks post‐booster. All pairwise tests were statistically significant. Antibody levels significantly increased from pre‐booster to 2 weeks post‐booster (median (25th, 75th percentile) from 59.94 BAU/ml (29.69, 177.8) to 6216 BAU/ml (3806, 11730)), corresponding to an average increase of 112 fold (Figure 1, Table 1). Antibody levels dropped to a median of 2654 BAU/ml (1650, 8340) 6 weeks post‐booster. This corresponded to a 36.3% average decrease compared with week 2. Between weeks 6 and 11, there was a 52.4% drop to a median of 1444 BAU/ml (1102, 2020). However, antibody levels at 11 weeks remained an average of 40‐fold higher than pre‐booster levels. Overall, antibody levels declined 47% month to month post‐booster.

**Table 1:** Humoral response of dialysis patients before (pre) and at an average of 2, 6, and 11 weeks after (post) a SARS‐COV‐2 BNT162b2 booster vaccine by COVID‐19 infection status

Timeline Median [Q1, Q3]^a^	Prior COVID infection	COVID naïve	Overall
	(*N* = 5)	(*N* = 22)	(*N* = 27)
6 weeks (pre)	1008 [230.7, 1037]	41.15 [23.33, 82.63]	59.94 [29.69, 177.8]
2 weeks (post)	3806 [1346, 6390]	6849 [4615, 12030]	6216 [3806, 11730]
6 weeks (post)	2423 [726.0, 4877]	3791 [1679, 8503]	2654 [1650, 8340]
11 weeks (post)	1452 [1118, 1670]	1444 [1102, 2178]	1444 [1102, 2020]

Abbreviations: COVID‐19, coronavirus disease 2019; SARS‐CoV‐2, severe acute respiratory syndrome coronavirus 2.

^a^ Median [25th quartile, 75th quartile] in BAU/ml.

Nine (33%) patients had negative or borderline detectable antibody levels pre‐booster and eight of nine developed positive (≥35.2 BAU/ml) antibody levels post‐booster. The one patient with negative antibody levels had never achieved positive antibody levels following two prior BNT162b2 doses. Notably, this patient was immune suppressed. Six patients had prior SARS‐CoV‐2 infection as identified by a Bio‐Rad Platelia SARS‐CoV‐2 total Ab assay assessing anti‐nucleocapsid IgG antibodies (Bio‐Rad Laboratories, Inc.). From pre‐booster to 2 weeks post‐booster, those with prior infection had a lower proportional increase in antibody level (51 fold) compared with the median change in COVID‐19 naïve patients (144 fold).

In earlier results, 40% of the cohort transitioned from positive to negative antibody status at 6 months post‐two‐dose vaccination.^5^ Encouragingly, a third dose appears to restore antibodies to high levels, though these waned quickly in the ensuing weeks. Similar trends of antibody decline are seen in healthy individuals, although dialysis patients may differ from the general population with reduced peak levels and lower seroconversion rates.^8^, ^9^ Long‐term durability remains unclear and protective levels against infection are unknown. Goldblatt et al.^10^ reported that the mean protective threshold against WT SARS‐CoV‐2 virus was 154 BAU/ml (95% CI 42–559) but higher levels are presumed to be required against current variants.

Interestingly, previously infected patients saw a blunted rise in antibody level after an initial booster shot, though these patients started from a higher baseline. Thus, overall, they attained similar peak levels. While our sample size precludes further analysis of this finding, the interaction of natural immunity with booster vaccination response in dialysis patients requires further study.

During the recent Omicron wave, boosters were found to be protective from hospitalization and severe illness in the general population, however, this effect was time‐dependent and declined significantly at 4 months post‐booster.^11^ A similar pattern is likely in patients on dialysis, but few studies have been conducted in this population. In one study, 93% of dialysis patients who received the third dose of BNT162b2 vaccine achieved antibody levels associated with protection, compared with only 35% pre‐booster.^12^ The Centers for Disease Control recommends a fourth dose of mRNA vaccines for select populations.^2^ The utility of such a strategy in dialysis patients remains unclear but the humoral antibody waning seen in dialysis populations may support additional boosters.

Our study has limitations: small sample size, brief follow‐up time and focus on humoral immunity. In conclusion, our data illustrate that, although humoral immunity wanes, patients on hemodialysis demonstrate strong antibody responses to a third dose of the BNT162b2 vaccine.

## AUTHOR CONTRIBUTIONS

**Eibhlin Goggins**: Conceptualization; investigation; methodology; writing – original draft; writing – review & editing. **Binu Sharma**: Formal analysis; software; visualization; writing – review & editing. **Jennie Z Ma**: Formal analysis; software; writing – review & editing. **Jitendra Gautam**: Data curation; investigation; methodology; writing – review & editing. **Brendan Bowman**: Conceptualization; funding acquisition; investigation; project administration; supervision; writing – review & editing.

## CONFLICT OF INTEREST

The authors declare no conflict of interest.

## ETHICS APPROVAL AND CONSENT TO PARTICIPATE

This research proposal was reviewed and approved by the University of Virginia Institutional Review Board for Health Sciences Research (tracking number: HSR 210095).

## TRANSPARENCY STATEMENT

The lead author Eibhlin Goggins affirms that this manuscript is an honest, accurate, and transparent account of the study being reported; that no important aspects of the study have been omitted; and that any discrepancies from the study as planned (and, if relevant, registered) have been explained.

## Data Availability

The data set used for this analysis is not publicly available. The data utilized was obtained from the Electronic Health Record and from the dialysis‐specific electronic medical record system, which is restricted to use by only authorized employees.
